# Stylistic variation across English translations of Chinese science fiction: Ken Liu versus ChatGPT

**DOI:** 10.3389/frai.2025.1576750

**Published:** 2025-06-19

**Authors:** Pingdi Zhou, Jiajun Cheng

**Affiliations:** South China University of Technology, Guangzhou, China

**Keywords:** science fiction translation, large language model translations, corpus-based translation, stylistic variation, Multi-Dimensional analysis

## Abstract

Advancements in computational tools, including neural machine translation (NMT) and large language models (LLMs), have revolutionized literary stylistics and opened new avenues in corpus-based translation studies (CBTS). Yet, the style of LLM-produced translations, especially in science fiction (SF) literature, remain understudied. This study examines stylistic variation across English translations of Chinese SF by translator Ken Liu and ChatGPT-4o. Thirteen works translated by both were compared using Multi-Dimensional analysis on key dimensions. Stylometric tests assessed within-translator and between-translator variations, and functional analysis interpreted the subordinate linguistic features. Findings reveal that Ken Liu adapts his style to each story’s depth, exhibiting greater variation, while GPT maintains a more consistent style. Ken Liu’s less narrative style enhances resonance through a minimalist approach, whereas GPT’s more narrative style offers clarity but may undermine thematic impact. The study contributes to CBTS by providing a methodological framework for comparing human and LLM translations in terms of style. It highlights a collaborative model that combines human creativity with LLM efficiency, necessitating continuous upskilling among students, educators, and practitioners to adapt to LLMs’ growing presence in translation. Ultimately, by exploring the intersection of linguistics, literature, and artificial intelligence, the study pushes the boundaries of translation studies and practices.

## 1 Introduction

Literary stylistics has increasingly embraced quantitative and computational methods, mirroring broader trends in linguistics. Advances in computational tools have enabled large-scale, empirical analyses, significantly advancing the field of corpus stylistics (Egbert, 2012). Building on these advancements, corpus stylistics has progressed to encompass detailed studies of stylistic variation, utilizing methods like Multi-Dimensional (henceforth MD) analysis to capture patterns and distinctions across genres, specifically speech and writing (Biber, 1991; Biber and Finegan, 1989). However, more recent work also emphasized the importance of variation in specialized genres. This would include Egbert’s (2012) seminal work on both within-author and between-author stylistic variation in 19th century fiction, a study on variation in L2 writing (Friginal and Weigle, 2014), and a book on linguistic features in TV shows (Quaglio, 2009), among others.

This focus has also evolved beyond creative literature. Baker (2000), the pioneer of corpus-based translation studies (henceforth CBTS), then applied corpus stylistics in the analysis of literature as translations, introducing “translator style” as a distinct and recognizable pattern of linguistic choices that uniquely identifies a translator across multiple works. Examples of corpus stylistics on translation include an investigation on translational Chinese genre system (Ji, 2016) and a study on orality features in translated and non-translated texts (Su and Liu, 2022), both of which utilized MD analysis. Only in very recent years have researchers begun comparing the stylistic features of works translated by different translators, including a study exploring differences between amateur and professional translations (Chou et al., 2024), and another examining styles of translators with different native languages (Gu and Chen, 2024), among others.

The advent of neural machine translation (NMT) and large language models (LLMs) unlocks even newer possibilities for the comparative study in CBTS. However, most studies are pivoted on evaluating quality or accuracy of translations done by generative artificial intelligence (henceforth AI), rather than examining style. For example, Ghassemiazghandi (2024) evaluated ChatGPT’s translation accuracy using the Bilingual Evaluation Understudy (BLEU) score, a metric in machine translation, while Jang and Lukasiewicz (2023) compared automated metrics and human judgement frameworks like MQM-DQF in evaluating LLM and NMT translations. Similarly, Karpinska and Iyyer (2023) demonstrated that LLMs can effectively use document-level context to improve literary translation quality, though their study, too, falls short of systematically investigating how stylistic elements are preserved across translations. Most notably among studies on the style of LLM translations was a study utilizing a hybrid approach that integrated statistical and algorithmic techniques with MD analysis to explore the boundaries of translations by human, NMT, and ChatGPT in translating spokesperson’s remarks, a type of diplomatic discourse within the non-literary genre (Jiang et al., 2023). More specifically, LLMs’ style in literary translations remains less extensively studied. One recent work by Resende and Hadley (2024) has begun exploring this area by examining how LLMs render stylistic features such as rhyme schemes, lexical richness, and sentence structure in poetry translation.

Despite these emerging efforts, research remains limited in scope, necessitating broader investigations into how LLMs perform across a wider range of literary genres and stylistic demands, especially given Ramírez Giraldo (2019) observed the way literary translation transmits the aesthetic properties and high cultural status to the target culture—a role that scholars argued LLMs are unsuitable to fulfil in translating literary works (e.g., Khoshafah, 2023). This complexity is further amplified in genres that rely heavily on imaginative and specialized language, such as science fiction (henceforth SF).

## 2 Previous research

### 2.1 SF, translation, and style

Historically, SF was dismissed by the literary establishment as juvenile and escapist (Attebery, 2003; Wolfe, 1986). However, from the 1960s onward, academic interest surged with the establishment of organizations such as the Science Fiction Research Association (SFRA) and the publication of critical surveys by authors like Ketterer (1971) and Suvin and Canavan (2016). The second wave of criticism incorporated feminist, Marxist, post-colonial, and post-modernist perspectives, broadening interpretative frameworks (Attebery, 1992; Luckhurst, 2005). In essence, SF serves as a powerful medium for social criticism and exploration of technological and societal changes (Suvin and Canavan, 2016; Freedman, 2000), thus its translation involves navigating “cognitive, linguistic, visual, cultural and ideological phenomena” inherent in the original work (Hatim and Munday, 2019). Moreover, the debate between “word-for-word” and “sense-for-sense” translation, dating back to Cicero and Horace (Bassnett, 2013), is particularly pertinent in SF translation within the context of LLMs, as their alignment with either approach compared to human translators is yet to be explored. Human translators, on the other hand, have expanded access for Anglophone readers and scholars in the last decade, by bringing diverse SF from other languages and cultures into English (Campbell, 2021). One of these translators that stands out is Ken Liu, a Chinese-American author and translator of SF, who famously translated *The Three Body Problem* by Liu Cixin, making it a best seller for Anglophone readers and a valuable academic resource. He has translated over fifty short stories and at least five novels from Chinese into English, introducing numerous Chinese SF authors to a global audience.

While great strides have been made in re-evaluating SF in terms of theme and socio-political significance, the study of style—as defined as the use of language for artistic function (Leech, 2007)—remained neglected, with prevailing opinions often viewing it as inadequate or unremarkable (Mandala, 2010). In *Science Fiction and Fantasy: Language, Style, and the Critics*, Mandala contended that style is not merely an ancillary feature but a fundamental component that enhances the imagination of future and past Englishes, presents the extraordinary as real, and constructs compelling characters. She emphasized a significant gap in scholarly research, advocating for a reassessment of style to fully appreciate the literary merits of SF. As such, understanding how Ken Liu’s style compares to that of LLMs would address the existing gap in CBTS, especially given the global popularity and the potential for cross-cultural dialogue of his SF translations.

### 2.2 MD analysis

MD analysis is a method for examining linguistic variation by analyzing the co-occurrence of linguistic features across multiple dimensions (Biber, 1991). MD studies are based on large corpora of naturally occurring texts that represent a range of registers within a discourse domain (Biber and Conrad, 2014). The first step involves analyzing the distribution of individual linguistic features within the corpus. Subsequently, exploratory factor analysis (EFA) or principal component analysis (PCA) is employed to identify systematic co-occurrence patterns among these features, referred to as “dimensions” (Goulart and Staples, 2023). Each dimension comprises a group of linguistic features that tend to co-occur in texts, such as nouns, attributive adjectives, and prepositional phrases, and these are interpreted qualitatively to assess their underlying functional associations (Biber and Conrad, 2014). Genre in MD analysis is defined by external factors like communicative purpose and situational context, which guide the classification and interpretation of linguistic patterns within texts (Nini, 2015; Biber and Conrad, 2019). For instance, specific linguistic features may co-occur in scientific reports to achieve procedural clarity, while others may dominate in narratives to emphasize storytelling (Gray, 2015). By focusing on these dimensions, MD analysis provides a structured framework for comparing texts and registers along each dimension, thereby describing overall patterns of register variation. This approach facilitates structured comparisons across genres or within specialized genres like literary fiction, as in this study.

Regarding stylistic research, Wu et al. (2024) corroborated that corpus methods in such research offers two main advantages: first, they enable the systematic analysis of frequency and distribution patterns of linguistic forms, providing statistical insights into stylistic patterns; second, they allow for the exploration of the literary functions associated with these patterns, linking linguistic description with literary analysis. Therefore, qualitative analysis with respect to the functional roles of stylistic patterns, referred to as functional style, adds credibility and robustness to a CBTS study.

However, issues related to LLMs, especially methodological ones, require careful consideration. For instance, Jiang et al. (2023) use of a single ChatGPT translation output may introduce a risk of arbitrariness due to inherent variability in AI-generated text. It has been proved that ChatGPT’s performance depends largely on settings such as “temperature,” which controls response randomness (Peng et al., 2023). Although varying temperatures can illustrate a broader range of stylistic variability, this study specifically seeks to evaluate the consistency of ChatGPT’s stylistic performance under a controlled condition. Thus, instead of altering the temperature setting, which would introduce an additional dimension of complexity, a constant temperature is maintained to perform multiple generation iterations for each text. This method effectively captures and stabilizes the typical stylistic tendencies of GPT at this given temperature setting, ensuring a consistent dataset for the comparative analyses.

Building upon Egbert’s (2012) empirical basis for examining within-and between-author variation using MD analysis, the present study aims to extend this to within-and between-translator variation. It explores the stylistic variation across English translations of Chinese SF by Ken Liu and GPT-4o, a multilingual, multimodal generative pre-trained transformer developed by OpenAI. The study further investigates how each translator addresses the translational complexities and aesthetics of SF, covering quantitative examination of linguistic features and their associated functional analyses.

## 3 Methodology

### 3.1 Corpus

The corpus of the present study comprises thirteen Chinese SF works from Ken Liu’s earliest translation collection, purchased from Amazon.com as e-books. Table 1 summarizes the basic information of the thirteen works. The Chinese source texts are available on Douban,^1^ a Chinese online database that allows registered users to record information and create content related to film, books, etc. Ken Liu’s translation of these works can be found on https://kenliu.name/translations/ and are carefully compiled into KL corpus (henceforth KL). Additionally, each Chinese text is translated by GPT-4o with the same prompt, “Please translate the following short fiction into English.” To mitigate potential coincidental or arbitrary variations, each text is generated by five separate runs (iterations), resulting in five sub-corpora of GPT (henceforth SCG). These stories explore diverse themes, offering substantial opportunities for examining both within-and between-translator variation. The former measures whether a translator’s style adapts to inherently different works, and the latter evaluates how potential re-occurrence of linguistic patterns distinguishes each translator even in varying contexts.

**Table 1:** Ken Liu’s SF translations.

Author	Chinese title	English translation and publication year	Token totals						Segment totals
			KL	G_1_	G_2_	G_3_	G_4_	G_5_	
Chen Qifan	《鼠年》	*The Year of the Rat, 2013*	9,312	12,379	12,146	12,029	11,876	11,806	74
	《丽江的鱼儿们》	*The Fish of Lijiang, 2011*	5,197	5,485	5,271	5,346	5,258	5,306	39
	《沙嘴之花》	*The Flower of Shazui, 2012*	5,923	5,315	4,981	5,053	4,990	5,190	46
Xia Jia	《百鬼夜行街》	*A Hundred Ghost Parade Tonight, 2012*	6,105	5,579	5,511	5,584	5,466	5,431	47
	《童童的夏天》	*Tongtong’s Summer, 2014*	5,718	5,173	5,060	4,997	4,916	4,970	43
	《龙马夜行》	*Night Journey of the Dragon-Horse, 2016*	5,249	4,634	4,960	4,653	4,747	4,641	39
Ma Boyong	《寂静之城》	*The City of Silence, 2011*	14,802	15,467	15,247	15,411	15,302	15,512	109
Hao Jingfan	《看不见的星球》	*Invisible Planets, 2013*	5,969	5,407	5,290	5,430	5,262	5,376	42
	《北京折叠》	*Folding Beijing, 2015*	15,956	14,720	14,186	13,980	13,823	14,024	101
Tang Fei	《黄色故事》	*Call Girl, 2013*	2,767	2,509	2,602	2,588	2,596	2,569	22
Cheng Jingbo	《萤火虫之墓》	*Grave of the Fireflies, 2014*	5,169	4,678	4,737	4,770	4,782	4,637	37
Liu Cixin	《圆》	*The Circle, 2014*	6,885	5,823	6,382	6,250	6,432	6,231	49
	《赡养上帝》	*Taking Care of God, 2012*	12,515	12,155	11,534	11,723	11,863	11,745	92
Total			101,567	99,324	97,907	97,814	97,313	97,438	740

The above works will henceforth be abbreviated as *Rat, Fish, Flower, Ghost, Summer, Journey, City, Planets, Beijing, Girl, Grave, Circle, and God*, respectively in the subsequent tests, tables, and figures.

### 3.2 Text segmentation and samples

This study combines a micro-segmenting method, which operates at the segment level, with the traditional chapter-level segmentation. Smaller segments enable more precise comparative examination of content, style, and linguistic features within manageable portions. Hearst (1997) supported this rationale, arguing that dividing text into finer-grained multi-paragraph segments (or subtopics) instead of broader sections improves information retrieval and facilitates detailed text analysis by creating more contextually meaningful units. Additionally, increasing the sample size within each work reduces the margin of error and enhances the reliability of results (Cohen, 2013). Text segmentation is executed using a Python (version 3.13) script, which processes text files first by chapters and then by segments, ensuring each segment contains least 100 words. When the word count reaches this limit, segmentation concludes at the end of the current paragraph, rather than stopping mid-sentence. With each new chapter, the segment numbering resets. For example, segments in Chapter 1 are labelled as 1_1, 1_2, and so on, while segments in Chapter 2 start again with 2_1, 2_2, etc. A final check ensures all segments meet the 100-word minimum, except for the last segment of chapters, which may be left shorter. This approach maintains both objectivity and granularity, enabling segment-to-segment comparisons. For this, GPT’s translations are segmented accordingly, meaning each segment can be directly matched with its corresponding segment from KL. This pairwise comparison establishes paired samples for this study, distinguishing it from many other studies (e.g., Jiang et al., 2023; Chou et al., 2024) that treated translations as independent samples. By treating segments as paired samples, the present study accounts for the shared content and structure, thereby allowing for a more accurate detection of potential differences attributed specifically to stylistic choices rather than varying text content or structure. The total segment numbers for each work can be seen in Table 1.

### 3.3 MD analysis of KL and SCG

In this study, MD analysis is conducted using Nini’s (2015) Multidimensional Analysis Tagger (MAT 1.3.3), a computational tool based on Biber’s model, to process the corpora. MAT has demonstrated strong accuracy in replicating Biber’s findings, enabling it to reliably capture linguistic variation. The tool employs six core dimensions that collectively describe patterns of co-variation among 67 linguistic features, representing key dimensions of English language variation (Nini, 2019). MAT first uses the Stanford Tagger for part-of-speech (POS) tagging, which allows for the precise identification of relevant linguistic features within the corpora (Toutanova et al., 2003). It then calculates corpus statistics based on the normalized frequencies per 100 tokens and dimension scores using standardized z-scores for linguistic features. All data for subsequent stylometric tests and their visualization are generated using R (version 4.4.2). Finally, the linguistic features results will be interpreted qualitatively regarding to their contextual functions.

Specifically, Dimension 1 contrasts the opposition between Involved and Informational discourse, where low scores indicate informationally dense texts, and Dimension 2 contrasts the Narrative and Non-Narrative concerns, where low scores indicate non-narrative texts (Nini, 2019). According to Biber (1991) and Biber and Conrad (2014) summary of text types, genres such as press reportage, press editorials, biographies, non-sports broadcasts, and SF are subsumed under the general narrative exposition text type, which typically get high score on D2, low score on D1, and unmarked scores for the other Dimensions. Empirically, Nini (2019) tested the generalizability of Biber’s model by applying the MAT to the LOB and Brown corpora—two structurally parallel datasets. This validation process reconfirmed that the core variation for general narrative exposition genres clusters overwhelmingly on D1 and D2. For instance, SF texts yielded strongly positive scores on D2 (around 4.79–6.10), reflecting a strong narrative focus, and moderately negative scores on Dimension 1 (around −4.10 to −5.01), indicating informational density. In contrast, D3-D6 displayed relatively minor or inconsistent variation, suggesting they are statistically less relevant for characterizing this text type under the MAT framework. Therefore, D1 and D2 serve as the focal point of this study.

### 3.4 Functional style

Functional analysis of translator style focuses on textual patterns, as defined by Hunston (2010) to mean meaningful repetitions of linguistic units like words and structures that carry different literary functions. Notable works by Mahlberg and McIntyre (2011), Mahlberg (2013), Čermáková (2015), and Mastropierro (2018) utilized keywords and key clusters, categorizing them into semantic domains that reflect various literary functions, such as the building of fictional world and fictional theme. This approach demonstrates that corpus stylistic studies can effectively explore the functional aspects of translator style in literary fiction, referred to as functional style (Wu et al., 2024). In this study, functional style will be explored under Wu’s theoretical framework. The procedure follows the MD analysis, examining linguistic idiosyncrasies, which is to analyze how each translator reproduce the stylistic patterns and recreate the original literary functions in their translations. It also involves investigating how the identified functional styles affect readers’ reception and interpretation of story elements such as themes, cultures, and worldviews. This step explores the relationship between translators’ stylistic choices and readers’ attitudes, providing insights into how translated works are received and promoted. This aligns with the ultimate goal of SF translation: to spark communication and ideas that transcend and resonate across national and cultural boundaries. Subsequently, researchers need to make sense of the findings by relating to translatorial factors (e.g., translator background, stance, preferences) and extra-translatorial factors (e.g., post-translation influence, patronage intervention) that may influence the development of translators’ styles.

### 3.5 Research questions

The study poses the following RQs:

1. Is there within-translator variation in KL and SCG across works and across SCG runs?
2. Is there between-translator variation?
3. What linguistic features define the observed stylistic patterns, and are these patterns dictated functionally by certain internal or external factors?

## 4 Results

### 4.1 Within-translator variation across works

MAT processed all the corpora, from KL to the five SCG throughout the thirteen works and output the dimension statistics. Table 2 contains dimension means and standard deviations for each of the thirteen works. Within each translator (KL, G_1_–G_5_), Kruskal–Wallis tests were conducted on segment-level dimension scores, grouped by the 13 translated stories (*df* = 12). Normality checks (Shapiro–Wilk tests) indicated violations of normality assumptions, justifying the use of the Kruskal–Wallis test as a non-parametric alternative to ANOVA. Consequently, six independent tests were performed on both dimensions—one per translator—each producing a *χ*^2^ statistic assessing within-translator variation across works.

**Table 2:** Means and standard deviations of the dimension scores.

Book	*M*						*SD*					
	KL	G_1_	G_2_	G_3_	G_4_	G_5_	KL	G_1_	G_2_	G_3_	G_4_	G_5_
Dimension 1												
*Rat*	7.91	6.64	5.51	7.10	5.99	6.45	9.86	10.12	10.30	11.19	10.32	11.02
*Fish*	9.11	4.87	4.39	5.12	4.81	5.25	11.00	11.00	11.38	12.08	12.09	11.76
*Flower*	1.58	−0.83	−1.65	−1.4	−1.47	−0.94	10.20	11.42	10.91	11.46	11.34	11.22
*Ghost*	3.71	−0.48	−0.93	0.43	−1.02	−0.52	9.06	9.78	9.51	10.08	9.59	9.25
*Summer*	4.38	3.50	4.65	4.04	3.83	3.62	8.70	7.75	7.07	7.47	7.91	7.97
*Journey*	1.00	0.07	0.84	−0.23	0.16	1.09	13.50	15.01	13.65	15.11	15.04	15.13
*City*	0.06	0.61	0.39	0.34	0.93	0.22	8.55	9.03	9.31	9.35	9.06	9.25
*Planets*	5.56	4.22	2.44	1.65	0.67	1.40	11.90	13.47	13.32	13.07	12.54	13.28
*Beijing*	1.06	−0.58	−0.29	−0.33	0.11	0.66	12.60	11.45	11.28	10.93	11.6	12.35
*Girl*	9.21	4.66	6.04	4.88	4.59	5.47	10.90	10.09	11.24	10.23	11.4	10.72
*Grave*	−2.02	−3.05	−3.13	−3.35	−3.21	−3.44	7.94	8.85	8.30	8.46	8.10	8.75
*Circle*	−0.36	−1.60	−2.9	−2.53	−1.95	−2.71	9.10	10.33	9.37	9.47	8.82	9.92
*God*	2.42	2.94	2.13	1.81	1.11	1.97	9.89	10.18	11.71	11.23	10.73	11.35
Dimension 2												
*Rat*	1.34	6.48	7.12	6.66	7.21	7.04	5.01	4.85	6.34	6.11	6.06	6.22
*Fish*	0.98	6.19	6.24	6.32	7.27	7.34	6.17	8.12	6.82	7.49	7.41	9.05
*Flower*	1.37	5.47	5.88	4.59	4.36	6.01	4.86	6.73	6.38	5.34	6.36	5.87
*Ghost*	0.64	7.65	9.29	7.37	9.15	9.83	4.37	7.11	6.80	7.01	8.36	7.80
*Summer*	4.03	6.98	7.27	7.06	7.84	7.95	5.97	5.73	5.95	5.92	5.73	5.19
*Journey*	1.50	5.68	6.04	8.23	6.97	7.00	5.13	6.09	6.87	7.25	6.86	7.09
*City*	3.97	6.47	6.84	6.90	7.57	6.94	5.39	5.62	6.58	5.74	6.20	5.85
*Planets*	0.90	3.33	4.62	3.69	3.69	3.43	5.62	7.89	7.20	6.54	7.52	7.03
*Beijing*	4.11	8.88	9.30	9.14	10.75	8.89	4.47	7.21	7.23	7.46	8.59	6.90
*Girl*	1.99	11.47	10.17	9.94	9.58	9.75	5.03	9.03	8.80	7.45	7.27	7.45
*Grave*	4.71	6.37	6.14	6.88	5.67	5.84	4.58	6.18	5.29	6.15	5.18	5.12
*Circle*	0.02	3.73	2.77	3.05	2.67	3.17	3.82	5.63	4.87	4.41	5.07	4.55
*God*	2.69	5.61	4.97	5.16	4.00	5.02	5.48	5.67	5.48	5.81	5.12	6.45

Table 3 presents the statistical results with effect sizes. On both dimensions, the *p*-values (< 0.0001) of KL and SCG invariably showed statistically significant differences, indicating within-translator variations exist across works in all six corpora. These variations within dictate that the thirteen works by one translator cannot be combined as one single corpus, but as separate entities in subsequent comparative tests, actually allowing for a granular analysis in which each work will be individually compared instead of being aggregated into a larger corpus.

**Table 3:** Kruskal–Wallis results for within-translators variations.

Corpus	Statistic	*p*	*ε* ^2^
Dimension 1			
KL	*χ*^***2***^(12) = 67.66	8.77e−10	0.08
G_1_	*χ*^***2***^(12) = 52.63	4.78e−07	0.06
G_2_	*χ*^***2***^(12) = 46.21	6.38e−06	0.05
G_3_	*χ*^***2***^(12) = 51.78	6.79e−07	0.05
G_4_	*χ*^***2***^(12) = 40.96	4.98e−05	0.04
G_5_	*χ*^***2***^(12) = 46.01	6.92e−06	0.05
Dimension 2			
KL	*χ*^***2***^(12) = 67.00	1.16e−09	0.08
G_1_	*χ*^***2***^(12) = 46.71	5.23e−06	0.04
G_2_	*χ*^***2***^(12) = 54.81	1.96e−07	0.06
G_3_	*χ*^***2***^(12) = 50.30	1.24e−06	0.05
G_4_	*χ*^***2***^(12) = 81.80	1.87e−12	0.10
G_5_	*χ*^***2***^(12) = 57.78	5.70e−08	0.06

Borrowed from Cohen’s benchmarks for η^2^, epsilon squared (ε^2^) effect sizes are often interpreted empirically as small (0.01), medium (0.06), and large (0.14). (Tomczak and Tomczak, 2014).

The Kruskal–Wallis *ε*^2^ statistic quantifies the proportion of variance in ranked data attributable to group differences. Theoretically, a higher *ε*^2^ indicates that stylistic differences among groups (translations in this context) explain a larger share of the variability observed, thus evidencing more pronounced stylistic divergence (Tomczak and Tomczak, 2014). The descriptive results of the effect sizes can be seen in Table 4. Across the five SCG, the *ε*^2^ values for Dimension 1 ranged from 0.0398 to 0.0559, with a mean of 0.0489 and low variability (13.3% coefficient of variation), indicating relatively consistent variation.

**Table 4:** Descriptive statistics for five runs of SCG *ε^2^.*

Book	M	SD	CV
Dimension 1	0.05	0.01	13.3%
Dimension 2	0.06	0.02	29.9%

In contrast, Ken Liu’s higher *ε^2^* value of 0.0766 suggested greater stylistic variation on this dimension. The results for Dimension 2, however, were more statistically interesting. SCG showed more variability on this dimension, with *ε^2^* values ranging from 0.0477 to 0.0960 (mean 0.0637, 29.9% variability). Although KL’s *ε*^2^ value (0.0757) falls within this range, it exceeds the average SCG *ε*^2^ (0.0637), indicating that KL demonstrated a generally higher level of within-translator variation on this dimension. These results suggest that, descriptively, Ken Liu demonstrated a higher level of within-translator variation across thirteen works on both dimensions, while ChatGPT maintains a relatively consistent style, especially on Dimension 1. The *χ^2^* values reinforced these findings, with *χ^2^*_KL_ value (67.66) being larger than all *χ^2^*_SCG_ values (ranging from 40.96 to 52.63) on Dimension 1, indicating KL’s greater variation. The same was for Dimension 2. The reason why KL demonstrated greater within-translator variation and variation along Dimension 2 exceeded that of Dimension 1 on the part of SCG, warrants further explanation.

### 4.2 Within-translator variation across SCG runs

Since statistically significant within-translator variation across works exist, this section focuses exclusively on SCG runs for each individual work, assessing whether consistent stylistic performance was maintained across runs. No comparison with KL is involved in this analysis.

Although the relative consistency within each SCG suggested by the descriptive statistics, this consistency across runs on each work could not be assumed. To confirm this, statistical tests were selected based on the structure and properties of the data. Specifically, since the five runs of SCG for each work are different translations of the same text segments, the data are paired rather than independent. Consequently, Friedman’s test (a non-parametric test for related samples) was employed when the normality assumption was violated, and Repeated Measures ANOVA was used when the normality assumption held. The paired nature of the data was determined by the direct correspondence of text segments across runs, ensuring that each translation run was structurally aligned. Normality was assessed for each comparison using the Shapiro–Wilk test. If normality was met, RM ANOVA was applied; otherwise, Friedman’s test was conducted. One-way ANOVA and Kruskal–Wallis tests, which assume independent groups, were deemed inappropriate for this context and thus not used.

Table 5 shows that on Dimension 1, two tests (on *Planet* and *God*) yielded *p*-values of 0.005774 and 0.0385, respectively. However, after applying the Bonferroni correction, the significance threshold is *α*_adjusted_ = 0.0038, calculated by dividing 0.05 by 13. Since these *p*-values exceed the adjusted threshold, the results are not statistically significant. The remaining tests on both dimensions produced *p*-values well above the threshold, confirming the consistency of the five runs of SCG.

**Table 5:** Friedman’s test and RM ANOVA results for SCG.

Book	Dimension 1		Dimension 2	
	Statistic	*p*	Statistic	*p*
*Rat*	*χ^2^*(4) = 5.26	0.26	*χ^2^*(4) = 1.37	0.85
*Fish*	*χ^2^*(4) = 1.43	0.84	*χ^2^*(4) = 2.66	0.62
*Flower*	*χ^2^*(4) = 1.50	0.83	*χ^2^*(4) = 9.06	0.06
*Ghost*	*χ^2^*(4) = 4.12	0.39	*χ^2^*(4) = 5.05	0.28
*Summer*	*F*(4, 168) = 0.59	0.67	*χ^2^*(4) = 0.26	0.99
*Journey*	*χ^2^*(4) = 7.37	0.12	*χ^2^*(4) = 8.04	0.09
*City*	*χ^2^*(4) = 2.27	0.69	*χ^2^*(4) = 6.81	0.15
*Planets*	*χ^2^*(4) = 14.53	0.01	*χ^2^*(4) = 8.58	0.07
*Beijing*	*χ^2^*(4) = 8.13	0.09	*χ^2^*(4) = 7.37	0.12
*Girl*	*χ^2^*(4) = 2.98	0.56	*χ^2^*(4) = 4.27	0.37
*Grave*	*χ^2^*(4) = 0.78	0.94	*χ^2^*(4) = 1.97	0.74
*Circle*	*χ^2^*(4) = 3.56	0.47	*F*(4, 192) = 0.66	0.62
*God*	*F*(4, 364) = 2.56	0.04	*χ^2^*(4) = 8.62	0.07

Given SCG’s consistency across runs, it would be unnecessary to treat the five sub-corpora individually and compare them one-by-one with KL, which is uneconomical in terms of resources and efficiency. Aggregating five runs of SCG, under this circumstance, would effectively reflect the overall performance of ChatGPT. It’s worth noting, however, that this aggregated corpus (henceforth AG) from SCG is non-substantial since texts themselves cannot be aggregated or averaged. For this, AG is an *artificial* corpus representing ChatGPT’s overall performance by averaging SCG’s dimension scores and linguistic feature scores. This averaging is both typical and methodical in that the influence of outliers is also minimized, while providing a more uniform and meaningful dataset for comparison. Additionally, as noted in studies on variationist linguistics (Nerbonne, 2008), aggregation methods are valuable in balancing the risks of noise, i.e., missing data, exceptions, and conflicting tendencies and inconsistencies, enabling a clearer and more reliable signal of linguistic trends.

### 4.3 Between-translator variation

As aforementioned, comparisons between KL and AG were only relevant when each work was treated individually, given the existence of within-translator variation across works. For each of the thirteen works, segment-level dimension scores from KL and AG were paired based on segment correspondence, resulting in paired sample structures. To determine the appropriate statistical test, the normality of the differences between paired samples was assessed using the Shapiro–Wilk test for each work and dimension separately. When the normality assumption was satisfied, a paired *t*-test was applied. When the normality assumption was violated, a Wilcoxon signed-rank test was used as a non-parametric alternative suited for paired, non-normally distributed data. This approach ensured that test selection was consistently aligned with both the paired nature of the samples and the underlying data distribution. As the tests were performed independently across thirteen works and two dimensions, potential multiple comparisons issues were addressed by applying a Bonferroni correction, adjusting the significance thresholds to *α*_adjusted_ = 0.00385 (*), 0.000769 (**), and 0.0000769 (***) respectively. Although the dimension scores included both positive and negative values, this posed no methodological concern—both the paired *t*-test and the Wilcoxon signed-rank test are designed to operate on differences that may span positive and negative ranges.

Therefore, thirteen paired *t*-tests or Wilcoxon signed-rank tests were performed to examine whether there were significant differences between KL and AG in both dimensions. Since the two types of effect sizes (Cohen’s *d* and Rank-Biserial *r*) obtained are not directly comparable, the works were ranked based on the percentage of segments showing a large effect size. As shown in Table 6, on Dimension 1, nine out of thirteen works exhibited no significant differences; on Dimension 2, twelve out of thirteen works exhibited significant negative differences. This finding strongly indicated that the style in KL was more non-narrative compared to AG, while the divergence with regard to involved and informational discourse was slight, with KL’s being modestly more affective and interactional. Since the dimension score differences in Dimension 1 were unmarked, the next section will revolve around the results on Dimension 2.

**Table 6:** Paired *t*-tests and Wilcoxon test results between KL and AG.

Book	Statistic	p	Effect size	%
Dimension 1				
*Girl*	*W* = 203.00	0.01	*r* = 0.60	63.64%
*Fish*	*t*(38) = 3.31	0.00	*d* = 0.38	53.85%
*Planets*	*t*(41) = 3.54	0.00	*d* = 0.28	52.38%
*Ghost*	*t*(46) = 4.49	4.72e-05	*d* = 0.46	51.06%
*Flower*	*t*(45) = 3.26	0.00	*d* = 0.27	47.83%
*Beijing*	*t*(100) = 2.22	0.03	*d* = 0.09	45.54%
*City*	*t*(108) = −0.76	0.45	*d* = −0.05	44.04%
*God*	*t*(91) = 0.61	0.54	*d* = 0.04	42.39%
*Journey*	*t*(38) = 0.70	0.49	*d* = 0.04	41.03%
*Circle*	*t*(48) = 2.91	0.01	*d* = 0.22	40.82%
*Summer*	*t*(42) = 0.45	0.65	*d* = 0.06	39.53%
*Rat*	*t*(73) = 1.76	0.08	*d* = 0.16	37.84%
*Grave*	*t*(36) = 1.40	0.17	*d* = 0.15	37.84%
Dimension 2				
*S*	*W* = 14.00	1.56e-12	*r* = −0.98	85.11%
*Flower*	*W* = 117.00	5.51e-07	*r* = −0.78	73.91%
*Girl*	*t*(21) = −7.13	4.93e-07	*d* = −1.26	72.73%
*Beijing*	*t*(100) = −10.21	3.37e-17	*d* = −0.93	63.37%
*God*	*Z* = −3.96	7.43e-05	*r* = −0.48	60.87%
*Rat*	*t*(73) = −7.59	8.11e-11	*d* = − 1.13	58.11%
*Journey*	*t*(38) = −5.77	1.19e-06	*d* = −0.95	56.41%
*Fish*	*t*(38) = −5.43	3.42e-06	*d* = −0.88	56.41%
*Circle*	*t*(48) = −5.25	3.43e-06	*d* = −0.81	53.06%
*Summer*	*t*(42) = − 3.33	1.83e-03	*d* = −0.62	51.16%
*City*	*t*(108) = −5.86	5.07e-08	*d* = −0.57	49.54%
*Planets*	*t*(41) = −3.8	4.66e-04	*d* = −0.45	45.24%
*Grave*	*t*(36) = −1.50	0.14	*d* = −0.31	40.54%

### 4.4 Factor analysis

According to Biber’s factor analysis, a general formula is (see Table 7). Dimension 2 = 0.90 (VBD) + 0.73 (TPP3) + 0.48 [PEAS] + 0.40 [PUBV] + 0.40 (SYNE) + 0.39 [PRESP] − 0.47 (VPRT) − 0.41 (JJ) − 0.34 [WZPAST] − 0.31 (AWL).

**Table 7:** Linguistic features in Dimension 2.

Linguistic feature	Abbreviation	Weight
Past tense verbs	VBD	0.90
Third person pronouns	TPP3	0.73
Perfect aspect verbs	[PEAS]	0.48
Public verbs	[PUBV]	0.43
Synthetic negation	SYNE	0.40
Present participial clauses	[PRESP]	0.39
Present tense verbs	VPRT	−0.47
Attributive adjectives	JJ	−0.41
Past participial WHIZ deletions	[WZPAST]	−0.34
Word length	AWL	−0.31

The numbers in front of the linguistic features on each factor are referred to as factor loadings or weights. They indicate the degree to which one can generalize from a given factor to an individual linguistic feature. The further from 0.00 a factor loading is, the more one can generalize from that factor to the linguistic feature in question. Thus, features with higher loadings are better representatives of the dimension underlying the factor, and when interpreting the nature of a dimension, the features with large loadings are given priority (Biber, 1991). For example, VBD will be one of the defining linguistic features in determining narrativity.

Biber (1991) also pointed out that the features with positive and negative weights on a factor have a special relationship to one another: the features with positive weights co-occur in texts, as do the features with negative weights. As such, these two groups of features occur in a largely complementary distribution. When a text contains several occurrences of features with negative weights, it will likely have few of the features with positive weights, and vice versa. Therefore, it can be suggested that works with the largest negative differences in narrativity used few features with positive weight on KL’s part, and were less influenced by the negative differences of negative-weighted features, if any, resulting in a pronounced negative difference between the KL (less narrative) and AG (more narrative). Conversely, works with negative differences but to a lesser degree, while also having few positive-weighted features on KL’s part, were relatively more influenced by the negative differences of negative-weighted features, resulting in a smaller negative difference in certain features between the KL (more narrative) and SCG (less narrative), which, subsequently “undermines” the overall negative difference. However, the reason for not positing works with smaller negative difference stem from a more frequent use of features with positive weight, thus making them more narrative, is that those features have higher loadings and are more likely to reverse the direction towards being more narrative, which contradicts with the actual findings. Works with negative differences undermined like *Summer*, *City* and *Planets*, with *d*-values of −0.62, −0.57, and −0.45, respectively (medium magnitude only), are presumably characterized either by more influential use of certain negative-weighted features, or simply by less pronounced negative differences produced by positive-weighted features. But Biber suggested that these two causes generally co-occur and complement each other.

## 5 Linguistic-functional analysis

### 5.1 Linguistic features differences

A paired *t*-test (or Wilcoxon signed-rank test) was conducted on the ten pairs of linguistic features between KL and SCG in each work. The statistical results for large effect size features that contribute and counteract can be seen separately in Table 8. Particularly, contributing features with the largest effect sizes are highlighted in green for paired *t*-tests and in orange for Wilcoxon tests. The asymmetry shown by the separate table illustrates the ultimate negative differences between KL and AG. Among the positive-weighted features that contributed, VBD, [PRESP], [PEAS], and [PUBV] were the most prominent (highlighted). The positive gap of VPRT, a negative-weighted feature with high loading, had a contributing effect in works with the largest negative differences, including *Ghost*, *Flower*, *Girl*, etc. Conversely, the negative gap of negative-weighted features like AWL and JJ, had a counteracting effect.

**Table 8:** Linguistic feature results that contribute and counteract.

Book	Linguistic feature	Statistic	*p*	Effect size
Contributing features				
*Rat*	[PRESP]	*Z* = −6.41	6.62e-09	*r* = −0.82
	VPRT	*Z* = 5.49	4.15e-08	*r = 0*.73
	VBD	*Z* = −4.24	2.24e-05	*r =* −0.57
*Fish*	[PEAS]	*Z* = −4.19	3.49e-03	*r* = −0.61
	VBD	*t*(38) = −12.79	2.40e-15	*d =* −3.04
	VPRT	*t*(38) = 15.50	5.14e-18	*d =* 2.38
*Flower*	VPRT	*Z* = 3.40	6.79e-04	*r = 0*.58
	[PRESP]	*t*(45) = −5.27	3.78e-06	*d =* −0.88
	VBD	*t*(45) = −4.67	2.70e-05	*d =* −0.80
*Ghost*	VBD	*Z* = −5.97	2.48e-09	*r =* −1.00
	[PRESP]	*Z* = −5.22	7.72e-07	*r =* −0.86
	[PUBV]	*Z* = −5.77	8.48e-04	*r =* −0.84
	VPRT	*t*(46) = 19.56	6.25e-24	*d =* 3.42
*Summer*	[PRESP]	*t*(42) = −4.41	6.98e-05	*d =* −0.97
*Journey*	VBD	*t*(38) = −7.69	2.92e-09	*d* = −1.82
	VPRT	*t*(38) = 9.23	2.99e-11	*d =* 1.18
	[PRESP]	*t*(38) = −4.92	1.73e-05	*d =* −0.84
*City*	[PRESP]	*t*(108) = −11.01	2.35e-19	*d =* −1.29
*Planet*	[PRESP]	*t*(41) = −4.71	2.82e-05	*d =* −0.93
*Beijing*	[PRESP]	*Z* = −8.28	9.86e-16	*r* = −0.94
*Girl*	VBD	*t*(21) = −13.71	6.03e-12	*d* = −3.62
	VPRT	*t*(21) = 12.03	6.94e-11	*t =* 2.61
	[PRESP]	*t*(21) = −5.47	1.99e-05	*d =* −1.37
*Circle*	[PUBV]	*Z* = −4.79	1.55e-03	*r* = −0.61
	[PRESP]	*t*(48) = −5.10	5.64e-06	*d =* −0.91
*God*	[PRESP]	*t*(91) = −6.99	4.36e-10	*d* = −0.93
Counteracting features				
*Rat*	[WZPAST]	*Z* = −6.48	6.99e-03	*r =* −0.50
	AWL	*t*(73) = −12.72	3.33e-20	*d =* −1.06
*Fish*	AWL	*t*(38) = −12.11	1.31e-14	*d =* −0.97
	JJ	*t*(38) = −4.90	1.80e-05	*d =* −0.91
*Flower*	AWL	*t*(45) = −10.69	6.23e-14	*d =* −0.82
*Ghost*	AWL	*Z* = −5.97	2.47e-09	*r =* −1.00
*City*	AWL	*Z* = −7.64	2.28e-14	*r* = −0.84
*Planet*	JJ	*Z* = −4.80	1.68e-06	*r* = −0.85
	AWL	*t*(41) = −10.62	2.41e-13	*d =* −0.85
*Girl*	AWL	*t*(21) = −5.88	7.78e-06	*d =* −1.02

### 5.2 Functional style

KL’s fewer uses of the positive-weighted features on narrativity played a major role in determining its gap with AG. After selecting the works in which these features differ the most and extracting one representative run from SCG, the features will be analyzed with regard to their functions.

Present participial clauses [PRESP] are one of the most contributing linguistic features in eight works, and differs with large effect size (|*d*| ≥ 0.80 or |*r*| ≥ 0.50) in eleven works. They are tagged when a punctuation mark is followed by a present participle (VBG), a preposition (PIN), determiner (DT, QUAN, CD), WH pronoun, pronoun (PRP), or adverb (RB) (Nini, 2015). The differences in [PRESP] are most defining in *Folding Beijing* (*r* = −0.94) and *The City of Silence* (*d =* −1.29), with Segment 1_23 in *Folding Beijing* having the largest difference (−3.87) among all others. This segment in KL and in G_1_ as a representative case is extracted for analysis.

*KL*: In the early dawn, the city folded and collapsed. The skyscrapers bowed submissively like the humblest servants until their heads touched their feet; then they broke again, folded again, and twisted their necks and arms, *stuffing* them into the gaps. The compacted blocks that used to be the skyscrapers shuffled and assembled into dense, gigantic Rubik’s Cubes that fell into a deep slumber. The ground then began to turn. Square by square, pieces of the earth flipped 180 degrees around an axis, *revealing* the buildings on the other side. The buildings unfolded and stood up, awakening like a herd of beasts under the grey-blue sky. The island that was the city settled in the orange sunlight, spread open, and stood still as misty grey clouds roiled around it. The truck drivers, tired and hungry, admired the endless cycle of urban renewal.

*G*_*1*:_ In the faint morning light, an entire city folded in on itself, *merging* into the ground. The tall buildings, like the humblest of servants, bowed low, *cutting* their bodies at the waist, heads meeting feet, *pressing* tightly together. Then they bent again, folding their heads and arms in, twisting and inserting themselves into the gaps. After bending, they reformed, *shrinking* into massive cubes packed tightly together, *falling* into a deep sleep. The ground flipped, section by section, *rotating* 180 degrees, *revealing* the buildings on the other side of the earth. The structures that had been lying folded beneath the surface stood up, rising like awakening beasts against the grey-blue sky. The city, like an island, settled into place, *expanding* outward, rising tall, enveloped in a misty grey cloud that hung in the orange morning light. The drivers, still tired and hungry, stood there, *witnessing* this endless, repeating urban theatre.

[PRESP] are considered as markers of narrative action, and are used for depictive discourse (Biber, 1991). They highlight actions in process and convey simultaneity or continuous action, often found in narrative fiction where the emphasis is on unfolding events. In the case of G_1_, the frequent use of PRESP such as merging, cutting, pressing, etc., creates a flow of actions featuring fluid and event-driven descriptions, while Ken Liu opts for finite verbs and conventional structures like “The skyscrapers bowed submissively… then they broke again, folded again,” focusing on completing one action before transitioning to the next.

In *Folding Beijing*, the author imagined a Beijing with a strict hierarchy ingrained in its design of folding transformations. The above excerpt is the first description of the folding process with informational density and abstract reflection. Ken Liu’s deliberate rendering punctuates and slows down the narration, drawing readers onto the reserved, cyclical, and mechanistic nature of the scene. By doing so, he successfully conveys the grandeur and complexity of the transformation and evokes a sense of ritual, mystery, and inevitability inherent in this story. On the other hand, G_1_’s version flows well and achieves formal equivalence and readability, showcasing its pursuit of faithfulness and correction in its rendering. The rest of the SCG share the same pattern.

Past tense verbs (VBD) are one of the most contributing linguistic features in five works, and differs with large effect size in seven works. On top of past tense verbs, this category also includes the conditional form of the verb to be (Santorini, 1990). Among the tests, VBD are the most influential factor in *Call Girl* (*d* = −3.62) and *One Hundred Ghost Parade Tonight* (*r* = −1.00). Specifically, Segment 7_1 in *Call Girl* has the largest difference (−12.48) and it is selected for analysis along with G_3_ as a representative.

*KL*: Six p.m. Rush hour. A tidal wave of humanity *emerges* from the subway stations, fills the shops, the roads, the overpasses. Xiaoyi *gets* out of the Charade. This is the world of the present. Dusk *burns* brightly and gently. Pedestrians *part* around her. Behind her is her shadow, stretched very long. Together, they *walk* slowly, with great effort. Xiaoyi *lifts* her hand to find the dog whistle hanging around her neck, *touches* it. They *exist*. They’ve always existed. She’s not alone at all. She *does* not cry.

*G_3_*: Six o’clock in the afternoon. The end of the workday, crowds *surged*, flowing over subway exits, shops, streets, and overpasses. Tang Xiaoyi *got* out of the little car. This *was* the real world, and dusk *burned* gently and brightly. The crowd *parted* as usual around her. Behind her *was* her shadow, stretched long, walking slowly and laboriously alongside her. Tang Xiaoyi *reached* for the whistle hanging around her neck. She *felt* it. They *were* all there; they *had* always been there. She *was* not lonely at all. She did not cry.

Narrative discourse depends heavily on past tense and perfect aspect verbs, presenting a sequential description of past events involving specific animate participants (Biber, 1991). The case in the G_3_ opts for past tense for all the verbs, framing actions as already happened and signaling a time-bound storyline. This contributes to the overall flow and engagement with the readers. In contrast, Ken Liu’s use of present tense phrases like “This is the world of the present,” “They exist,” and “She does not cry” deviates from conventional narrativity, set in the present moment in an observational and ethereal manner, rather than a simply sequential one.

In *Call Girl*, the line between reality and surreality is blurred in that the girl’s detachment from society clashes with her supernatural power. It explores the theme of escape from emotional and societal entrapment in pursuit of a utopian existence. Ken Liu’s intentional use of present tense effectively delivers the blurred temporal realities—past, present, and future—immersing readers in a sense of immediacy and timelessness, as depicted in the work. This aptly aligns to the philosophical reflection and existential themes of the work, instead of a task-driven accuracy by clear temporal progression, sequential storytelling, and actions themselves prioritized by SCG. While the one-size-fits-all use of VBD on SCG’s part is safer, simpler, and widely applicable, it fails to render the more context-based flair inherent in SF’s tendency to explore broader concepts such as reality, existence, and time. All the remaining sub-corpora use VBD.

Present tense verbs (VPRT) are shared by two works as one of the most contributing linguistic features and differ with large effect size in six works. Any verb that received by the Stanford Tagger a VBP or VBZ tag (present tense or third person present verb) is tagged as VPRT (Nini, 2015). The most prominent differences of VPRT are represented by *One Hundred Ghost Parade Tonight* (*d* = 3.42) and *The Flower of Shazui* (*r = 0*.58). The positive effect size values also manifest that VPRT is one of the only two features that have large negative weight on being a narrative discourse (Biber, 1991). Since the dichotomy between VBD and VPRT has already been discussed above, case analysis on specific segments pertaining to VPRT will be omitted. However, it’s worth noting that, very similar to *Call Girl*, *One Hundred Ghost Parade Tonight* also blends SF with elements of fantasy and the supernatural, exploring themes of identity, mortality, and the impermanence of things. The story is filled with both wonder and a sense of melancholy as it explores the boundaries between life and death, reality and illusion. This well justifies Ken Liu’s resort to VPRT in his translation, which again emphasizes that the characters’ experiences are not just in the past but part of an ongoing, ever-present reality, in line with the motif of the source text. Readers are therefore inevitably invited to reflect on what it means to be real or human in a world increasingly shaped by artificiality right at the present day, instead of simply on the plot of an event-driven narrative that unfolds naturally and rationally. This choice is further supported by Harvey (2006), who argued that VPRT disrupts traditional narrative order, reflecting the chaotic reality of contemporary life, offering a “now” perspective that enhances reader engagement.

Public verbs [PUBV] is one of the most contributing features in one work, and differs in two with large effect size. It is tagged when verbs involved communication, like acknowledge, admit, agree, etc. are found (Quirk et al., 1985). The differences in [PUBV] are most pronounced in *The Circle* (*r* = −0.61), with Segment 2_9 having the largest difference (−2.18). G_1_ is selected for analysis.

KL: “Never?” “Yes. Imagine a silk cloth as large as all-under-heaven. The string of numbers in the circle’s ratio could be *written* in tiny script, each numeral no bigger than the head of a fly, all the way from here to the edge of the sky, and then coming back here start on a new line. Continued this way, the entire cloth could be filled, and there would still be no end to the numbers, and the sequence still would not…

G_1_: “Never *repeating*?” “Yes. Imagine a giant piece of silk as large as the world. This number could be *written* in the tiniest script, from here all the way to the horizon. Then you could start a new line and continue *writing* until the entire world is covered, and still, no part of the sequence would…

According to Biber (1991), public verbs, also as markers of narrative action, are used frequently for reported speech (e.g., admit, assert, declare, hint, report, say). They emphasize actions and statements within a narrative, often connecting the narrative voice with the dialogue or thoughts of characters and creating a sense of storytelling or reporting. As in the case of G_1_, phrases “written in the tiniest script” and “writing until the entire world is covered” describe a very clear process within the dialogue, connecting the imagined action and the narrative voice by using the public verbs “written” and “writing.” However, Ken Liu’s use of only one public verb (“written”) and the avoidance of another (“continued this way” rather than “continuing writing”) makes the dialogue in question more abstract. The same holds true for the omission of “repeating,” which creates a conceptual distance and intangibility of the idea by the stark simplicity of “Never?” Symbolism permeates *The Circle*. The “circle” represents both mathematical complexity and an unreachable ideal, symbolizing the ultimate truths of the universe. And the idea of calculating *π* becomes a metaphor for humanity’s desire to grasp what lies beyond mortal understanding. These terms are used abstractly to explore the theme of humanity’s reckless pursuit of godlike power, and warn against the aftermath metaphorically. Ken Liu’s sparse use of public verbs once again marks his retrospective approach, favoring contemplation over mere action. This not only aligns with the enigmatic, allegorical style of this story, but with the nature of SF at large. In contrast, G_1_’s version guarantees the clarity and relatability of the mathematical concept being introduced, but underplays the philosophical weight and the thematic depth found in the original text. This evidences GPT’s focus on precision and factuality, prioritizing error-free delivery over stylistic adaptation.

Likewise, perfect aspect verbs [PEAS] are also used to refer to actions in the past (Biber, 1991). A simple example is provided below. The following excerpt is taken from *The Fish of Lijiang* (segment 2_1, with the largest difference of −2.65), where [PEAS] act as the most differing feature in the Wilcoxon test (*r* = −0.61).

KL: …After ten years, everything here *has* changed. The only thing that remains the same is the color of the sky.

Lijiang, I’m back. This time, I’m a sick man.

G_3_: …and ten years *had* passed. Everything that should have changed had changed, except for the color of this sky.

Lijiang, I’m back. This time, I am a patient.

In G_3_’s version, the frequent use of [PEAS] establishes a temporal sequence that emphasizes progression and transformation over time, while KL’s minimalist technique again adds a timeless and thought-provoking quality, breaking the cohesive narrative sequence inconsistent with the themes of authenticity, temporality, and the search for meaning within an artificial, hyper-commercialized world in the story.

Biber (1991) contended that the interpretations of the factors, both the negative and positive cluster of features, must be taken into consideration. An example was given as follows: When past tense, third person pronouns, and perfect aspect verbs occur with a high frequency in a text, present tense verbs and adjectives are likely to be notably absent from that text, and vice versa. This has been confirmed in the present study as negative-weighted features like VPRT were prominently present when great negative differences were found in VBD and [PRESP]. This distribution contributes to KL’s overall non-narrativity compared to AG. In theory, non-narrative texts are expected to show a positive difference in negative-weighted features. However, if a non-narrative text exhibits a negative difference in these features, which would, to some extent, counteract its non-narrativity, it still supports Biber’s expectations regarding distribution trends when that negative difference is smaller than in a less non-narrative text. Specifically, in the case of *Ghost*, the most non-narrative work compared to GA, although its non-narrativity is counteracted by negative differences in AWL, this negative difference is smaller than that in differences in *Plants*, the most narrative work compared to GA, where both AWL and JJ undermined its non-narrativity (see Table 8). It can be understood, therefore, that Biber’s theory focuses on the relative distribution of linguistic features rather than their absolute values. This addresses the postulation proposed at the end of the stylometric tests. Since AWL is the only re-occurring linguistic feature that renders KL’s style slightly more narrative, it will be discussed below.

Word length has minimally salient loading (−0.31) as per Biber’s (1991) computation, which explains its slight counteracting effect. However, AWL is one of the most prominent features in all the seven works that contain counteracting features with large effect size. Understanding the way KL’s shorter word length distinguishes his style from that of AG underscores a subtlety commonly unnoticed. AWL differs most in *The Year of the Rat* (*d* = −1.06), with Segment 10_1 having the largest difference (−0.746).

KL: Pea finally said something meaningful. “Living is so …”

He did not finish his sentence. Tiring? Good? Stupid? You could fill it in however you wanted. That was why I said it was meaningful. Compared to his old way of talking, this new style was forceful, to the point, and left plenty of room for imagination. I admit it—all those literary criticism classes did teach me something.

For me, living was so … unbelievable. Half a year ago, I never imagined that I would get to bathe only once a week, that I’d be sleeping with lice in the mud, that I’d fight other men my age for a few stale wowotou biscuits, that I’d tremble with excitement at the sight of blood.

G_2_: Pea finally said something insightful. He said, “Living is really fucking…”

What was he really fucking? He did not say. Was it really fucking exhausting, really fucking refreshing, really fucking meaningless? You could fill in any word you wanted, which is why I said it was insightful. Compared to his previous long, flowery sentences filled with redundant parallel structures, this sentence was short and powerful, leaving endless room for imagination. Okay, I admit that the literary criticism class did teach me some things.

For me, living is really fucking incredible. I mean, 6 months ago, I could never have imagined showering once a week, sleeping in the mud with bedbugs, fighting over spoiled corn buns, climbing a mountain 1 day and another the next, and getting excited just by seeing blood.

Word length marks a high density of information, by reflecting precise word choice and an exact presentation of informational content. Longer words tend to be rarer and more specific in meaning than shorter words (Biber, 1991). G_2_’s use of lengthier words and phrases, such as “insightful,” “meaningless,” and “redundant parallel structures,” and the inclusion of several swear words naturally add to the overall word length and syllable count, introducing an evaluative and descriptive tone. In contrast, KL’s version employs shorter words like “good,” “stupid,” “unbelievable,” and “fight,” contributing to a more concise and direct narrative.

*Rat* delves into themes of survival, identity, and the ethical dilemmas of human intervention in nature, warning against the repercussions of turning nature and humans alike into instruments of industry. In the story, the protagonist’s disillusionment unfolds gradually, in stages, rather than all at once. To this end, KL’s lexical simplicity facilitates readers’ engagements and leaves “plenty of room for imagination.” This style fills in the thematic and emotional gaps present in his SF translations, where abstract or speculative ideas abound. On top of accessibility, this brevity makes each word carry more weight and impact, and would in effect, elicit stronger emotional responses, in turn matching the philosophical depth of the story. On the other hand, G_2_’s longer and more complex expressions adds to the descriptive weight, yet resulting in a more controlled and safe style that distinguishes itself from KL in the skill of harmonizing clarity with profundity. Other SCG all exhibit similar patterns, suggesting an absence of tendency for GPT to adopt a minimalist approach characterized by short and potent words that impress.

## 6 Discussion

### 6.1 Interpretation of findings

KL’s larger effect sizes in the Kruskal–Wallis test on both dimensions reveal a greater within-translator variation, reflecting his adaptability to genre-specific conventions. This aligns with the notion that, in literature, particularly in SF, credibility is achieved through coherence within genre-specific reading protocols rather than mere logical consistency (Tuzet, 2022; Noletto, 2024), as evidenced by SCG’s smaller effect sizes. In SF, authors and translators often employ what Tuzet described as a “disguised arbitrariness,” a façade of rigorous scientific logic rendered in prosaic style, to make fantastical elements appear plausible and authentic to the reader. This technique creates a central “poetic illusion” that invites readers to suspend disbelief and engage deeply with the narrative’s speculative aspects. As Noletto explained:

“*For instance, a realistic novel might have to maintain closer proximity to the reader’s actual world coherence than a speculative text in order to be credible or engaging. This emulated proximity might involve a sort of disguised arbitrariness typical of the SF genre—a façade of rigorous scientific logic rendered in prosaic style—the central poetic illusion, or a simple appearance of plausibility and authenticity, a realistic representation of fantastic things* (Csicsery-Ronay, 2012).”

Cognitively, the “poetic illusion” operates through the principle of minimal departure (Ryan, 1980), allowing readers to integrate speculative concepts into their real-world schemata, thus facilitating deeper immersion. In the case of this study, this illusion can be exemplified in *Folding Beijing*, where Ken Liu trades off syntactical logic for cognitive coherence under the genre’s reading protocols. He constructs this illusion through the serious technical description of the city’s transformation. This sublime or even overpowering process creates an illusion that departs minimally from the cognition of imposing architecture reminiscent of real-world scenes. The façade of rigorous technical logic*—*a “disguised arbitrariness”— reduces the cognitive dissonance that might arise from the implausibility of the concept of a folding city, making it seem credible to the reader but resulting in different stylistic choices (e.g., the avoidance of [PRESP] to slow down the narration in this case) in its rendering. This aligns with the notion that such an illusion evokes curiosity and wonder while maintaining a balance between familiarity and estrangement (Noletto, 2024), i.e., a real-world scene and unconventional syntactical structure in this case. Similarity, Freud (2017) used the term “unheimlich” (uncanny) to refer to this readerly effect. By adapting his translation to the specific demands of each story’s framework, Ken Liu creates such readerly effect characteristic of SF’s successful constructs, leading to significant stylistic variation across different works.

In contrast, SCG’s smaller variation suggests a tendency towards logical consistency and factual accuracy in its translation, prioritizing literal translation over adapting to genre-specific conventions that aid in creating the poetic illusion necessary for SF. Such a priority is critiqued by Eugene Nida (1964), who proposed functional equivalence to outline a readerly effect as well. This equivalence of response emphasizes that the target audience should react to the translation in a manner similar to how the original audience responded to the source text, also aiming to balance the familiar and the estranged. In this case, GPT’s uniform translator style presented the speculative elements with direct logic and authenticity (i.e., the natural flow of the city’s transformation in this case) without considering the need for eliciting the same sense of sublimity of that process. This can paradoxically make the fantastical seem less credible and impactful within the genre’s expectations. This is further supported by two empirical studies (Gao et al., 2024; Chu and Liu, 2024). Gao’s study demonstrated GPT’s proficiency in translating linguistic elements of Chinese classical poetry. This proficiency contributes to its logical and consistent translations. Chu suggested that while GPT’s linguistic competence and logical coherence contribute to its narration, its overall storytelling ability may be limited by a lack of lived experience and creativity. This limitation is also highlighted by the present study, which revealed that GPT’s logical and consistent translations per se are less impactful to the reader. Similarly, SCG’s smaller variation on D1 compared to D2 (see Table 4) may also exemplify its tendency to deliver informationally faithful translations, with D1 being the spectrum of informational density and affectivity.

**Figure 1:**
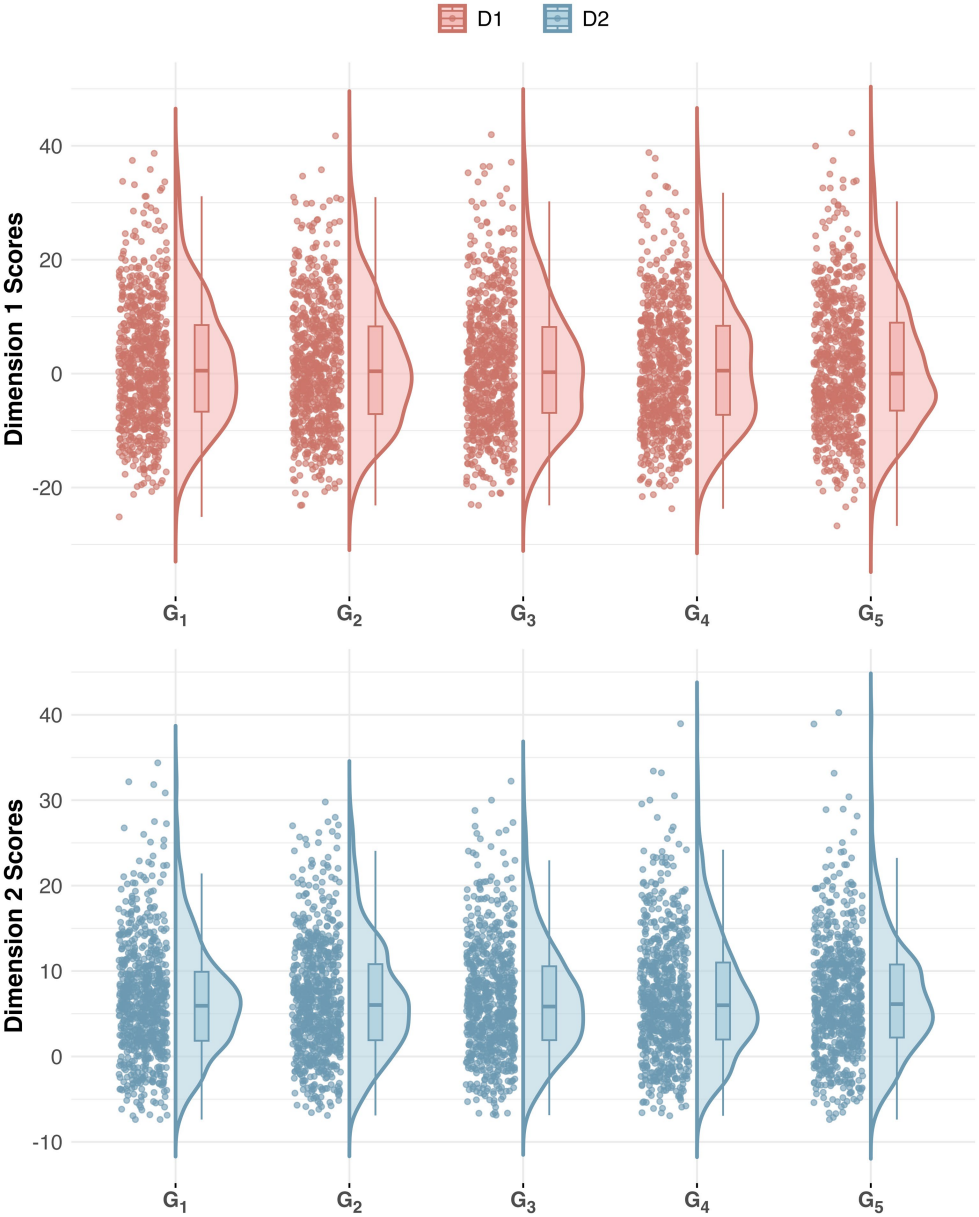
Iteration consistency. The half-violin plot with jittered pointes visualizes within-translator variation across SCG iterations with both smoothed density distribution and exact values.

Large *p*-values in Table 5 and the consistent shape of the half-violin plots as well as the box plots inside (see Figure 1) validate the absence of within-translator variation across runs in SCG, which has not been explicitly covered in existing literature. In fact, some studies have suggested that GPT frequently fails to demonstrate trustworthiness regarding logically consistent behavior (e.g., Jang and Lukasiewicz, 2023). However, it’s worth noting that these findings are based on diverse downstream tasks rather than style in translation. The present study’s use of structured and controlled prompts likely accounts for the contrasting results, emphasizing the role of task-specific parameters and prompt design in influencing performance. Practically, this discrepancy indicates that claims of GPT’s inconsistency might be overly generalized, highlighting the need for re-evaluating its capabilities especially in domains where stylistic consistency is critical. For instance, in pedagogical settings, students should aim to produce texts that are consistent and coherent, instead of striving for an unattainable “ideal” translation model characterized by exact equivalence (Davies, 2004). This reinforces the importance of task-specific evaluation, where GPT’s translations can serve as reliable demonstrations aligned with pedagogical objectives, rather than a flawless panacea to all logical challenges. Academically, the consistency observed can also serve as a preliminary reference for future research on the comparative stylistics of LLM translations, thereby avoiding potentially resource-intensive iterations. These scenarios demonstrate instances where LLM’s consistent translator style might be preferable despite current imperfections in literary translation or advanced tasks.

**Figure 2:**
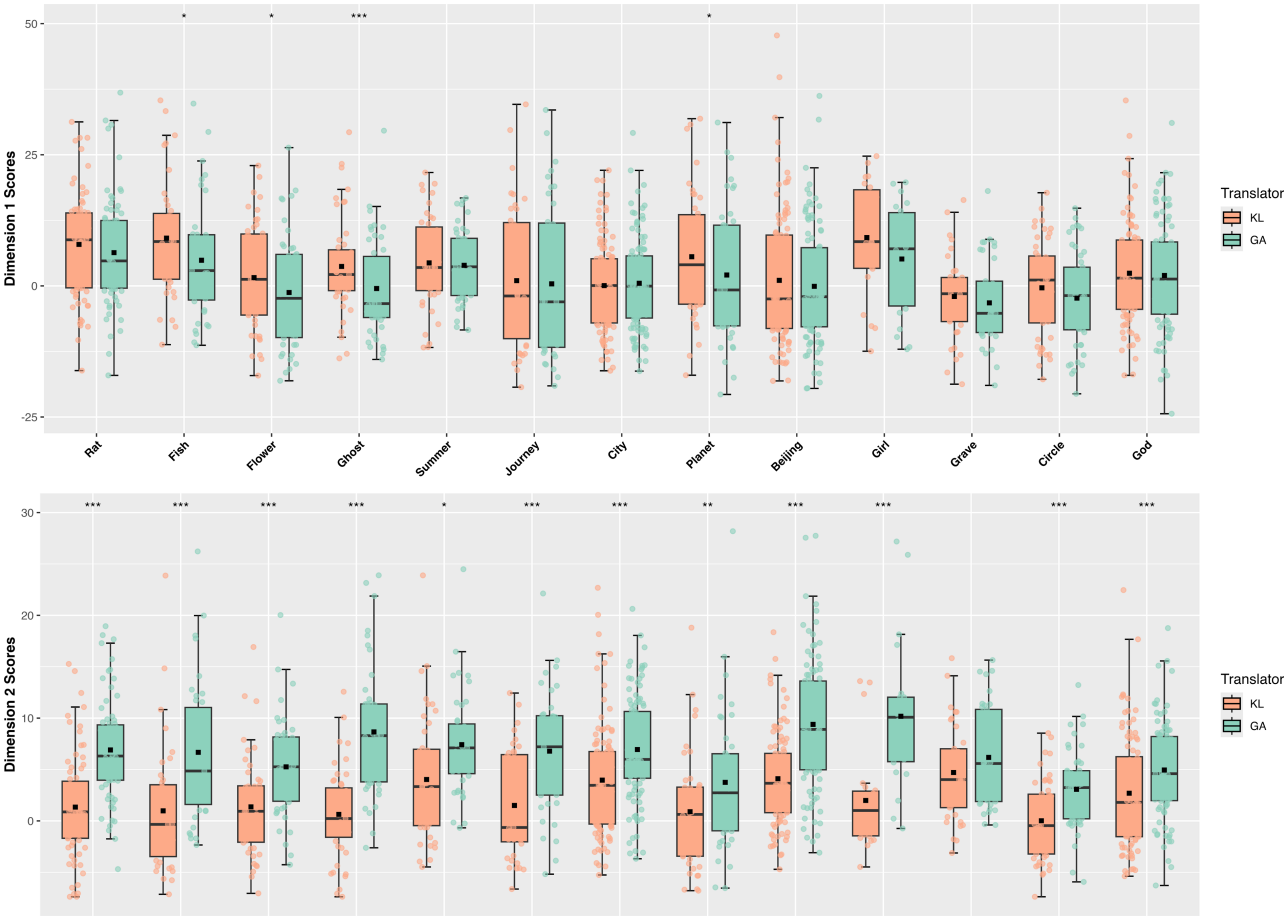
Between-translator variation. The boxplot above visualizes the KL and GA’s general performance on both Dimensions, from which marked differences can be observed in D2.

The subsequent between-translator variation examination (see Figure 2) reflected unmarked differences between KL and AG in D1, with KL being slightly more stylistically involved and affective, as opposed to being more informationally dense. These two findings reinforce each other. On the one hand, GPT’s translational tendency towards logical consistency and factual accuracy with the source text resulted in no significant differences from Ken Liu in terms of information conveyance, as measured by D1. On the other hand, this same tendency rendered GPT’s translations modestly more informational and less interactional compared to Ken Liu’s. In terms of D2, the negative differences varying in degree can be attributed to the varying themes the works explore. Works involving dystopian worlds and existential struggles such as *Ghost*, *Flower*, *Girl*, and *Beijing*, as analyzed, are translated by Ken Liu with a less narrative style. Conversely, stories with rich imagery and allegory that prioritize storytelling such as *Grave*, *City*, and *Planets*, are translated with a more narrative style.

The study then found that linguistic features such as VBD, [PRESP], and PUBV, emerged as key stylistic makers in determining the negative differences on narrativity between KL and AG. In summary, KL’s style is minimalist, abstract, and contemplative. This selected approach enhances thematic depth, philosophical reflection, and emotional resonance. In contrast, AG’s style is detailed, descriptive, and event-driven. This ensures clarity, readability, and formal equivalence, maintaining a straightforward narrative flow. Liu’s skillful use of these features invites contemplation, while GPT’s controlled approach prioritizes precision and accessibility, reflecting two distinct aesthetic ideals in literary translation. These findings corroborate with broader discourse on AI integration into CBST, as experimentally explored by Tekwa (2024). The study observed that AI-generated translations sometimes exhibit excessive literalness, where phrases or sentences are translated word-for-word, especially for complex or idiomatic expressions. The study further notes that while generative AI models can expedite the translation process by delivering more accurate initial outputs, they may also introduce issues like tense inaccuracies. This reinforces the present study’s finding of AG’s indiscriminate use of VBD within the context of literary fiction.

Following linguistic idiosyncrasies, the present studies then analyzed their functional role and revealed that Ken Liu’s minimalist and contemplative approach effectively delivers the thematic and emotional complexities that GPT’s error-free approach finds challenging to achieve. Specifically, Ken Liu’s style echoes a reductionist or minimalist approach typical of Ernest Hemingway, who utilizes the surface elements of an “iceberg” to reveal deeper emotional and thematic undercurrents through linguistic directness and simplicity (Levin, 1951). The divergences between the two translators’ functional style can be attributed to a combination of translatorial and extra-translatorial factors (Wu et al., 2024). From a translatorial aspect, Ken Liu’s dual role as a writer and translator of speculative fiction, along with his familiarity with the genre’s thematic preoccupations, such as existentialism, reality versus illusion, and societal critique, guides his stylistic choices. These choices exemplify the human habitus and creativity in literary translation, where the translator’s unique blend of cultural, cognitive, and creative skills, which developed over time, enables the capture of the text’s literary qualities (Wang, 2023). In terms of extra-translatorial factors, Liu operates within a publishing context that likely supports and encourages his creative stylistic decisions. Collaborations with editors and publishers who value literary innovation may provide him with the freedom to deviate from conventional translation practices in favor of preserving the original’s artistic integrity. This aligns with the concept of Actor-Network Theory (ANT) by Latour and Callon and multi-actor interactivity, as discussed by Wang. According to ANT, literary translation is a complex network of interactions among human and non-human actors (e.g., translators, editors, publishers, and tools). Liu’s work exemplifies how this collaborative network contributes to producing meaningful translations. Culturally, Liu’s bicultural identity positions him to make stylistic choices that bridge cultural gaps between Chinese and Western literary traditions, enhancing the accessibility and appeal of Chinese speculative fiction to English-speaking audiences. In contrast, LLM translations, while proficient in linguistic accuracy, lack the ability to intentionally incorporate such complex actor considerations. From a quasi-human actor perspective, GPT’s emphasis on conventional narrative structures and readability reflects its programming priorities rather than a deliberate stylistic stance. Lacking personal experiences or cultural intuition, LLM translations may prioritize literal correctness over thematic depth, resulting in versions that are clear but potentially less evocative and impactful. Culturally, this literal correctness may also reflect itself as a deeper cultural and political one, as noted by Cao et al. (2023), who found that GPT, a multilingual model, paradoxically aligns strongly with American cultural norms—contrasting with the bicultural competence of Ken Liu, who is also an American. This alignment, likely due to the dominance of English-language training data, renders GPT’s adaptability to other cultural contexts, such as Chinese, Japanese, German, and Spanish, less effective (Cao et al., 2023). This limitation potentially inhibits its ability to capture the stylistic and cultural conventions of SF, a form of significant cultural practice (Weldes, 2003). Weldes argues that SF, through its intertextuality with world politics, not only reflects and critiques ideologies but also actively shapes cultural understandings of power, sovereignty, and resistance while offering alternative visions of societal and technological futures. Therefore, translating SF requires more than just linguistic proficiency; it demands an unbiased cultural standing. In other words, this socio-political complexity warrants human supervision, intervention and collaboration in translating SF and other genres with similar pursuits. This partnership will counterbalance the impact of LLM’s inherent absence of extra-translatorial factors and non-human actors in practice.

### 6.2 Limitation and future research

This study presents three limitations, which are also relevant to other large-scale, linguistic analyses of stylistic variation. First, since this comparative study aims to investigate stylistic variation between a human translator and a LLM as it is, rather than a LLM intervened, experiments validating its performance after interaction are beyond the scope of this research. While it can be well anticipated that as newer and more advanced models spring up, human instructions, insofar as they are based on reasoning, will be processed more effectively and efficiently, future research is needed to confirm this. Such research should also explore prompt engineering in translation tasks for literary fictions, and more genres, such as poetry, drama, or non-fiction. Second, stylistic variation encompasses more than just grammatical and lexicogrammatical differences. Many facets of literary style, including semantics, rhetorical devices, and cultural references, cannot be effectively captured through quantitative methods like MD analysis alone. Approaches such Machine Learning Classifiers, Natural Language Processing (NLP) techniques, Knowledge Graphs, etc. are all state of the art in the field of literary stylistics and can be incorporated to comprehensively examine literary style. Third, due to length constraints, the evaluation did not incorporate reader reception studies or assessments of the translations’ impact on actual readers. In future research, such endeavor could provide empirical support for the effects of stylistic differences on reader engagement and interpretation.

## 7 Conclusion

This is the first study addressing stylistic variation between human and large language model (LLM) translations in the domain of science fiction (SF). By employing Multi-Dimensional (MD) analysis and functional stylistic analysis, it revealed both within-and between-translator variations. Ken Liu’s translations exhibit a greater stylistic adaptability and are characterized by a minimalist, contemplative approach that aligns with the thematic and philosophical depth of the source texts. This results in less narrative discourse. In contrast, GPT’s translations are consistent and coherent but fall short of the stylistic adaptability needed to fully capture genre-specific subtleties. The LLM’s focus on clarity and readability results in more narrative and event-driven style, but one that is less thematically credible and impactful. These findings emphasize the crucial role of human translators in literary translation, particularly in genres that rely heavily on imaginative and philosophical exploration.

This study carries several implications for the field of corpus-based translation studies (CBTS) and beyond. Firstly, the observed stylistic variation highlights the necessity of incorporating both human and LLMs in comparative stylistic research. This dual analysis deepens the understanding of how human creativity and machine logic manifest differently in literary translation and how they can complement each other. However, this collaboration inevitably carries ethical implications. The rapid iteration of LLMs risks diminishing the role of human translators in the publishing industry and education, potentially impacting job opportunities and the professional standing of translators. This situation propels students, educators, and practitioners alike to engage in continuous learning and upskilling (e.g., literary competence, cross-cultural understanding, AI tool utilization, etc.) to adapt to the growing presence of AI in the industry. Secondly, while LLMs like GPT-4o excel in producing translations that are linguistically accurate and stylistically consistent, they lack, in their current form, the capacity for the complex interplay of stylistic and cultural elements that contribute to thematic depth and emotional resonance. Understanding these limitations will guide future collaborative models in translation practice, where human translators first evaluate thematic elements of the work in question and design tailored prompts to address initial stylistic issues, such as tense choice, before cross-referencing cultural viewpoints and finalizing translations. By synchronizing LLM efficiency with human expertise, the translation process can be largely enhanced. Furthermore, this collaborative model will constantly generate user interaction data for fine-tuning LLMs, a process where pre-trained models are optimized based on user feedback. Developers should also incorporate training data that reflects diverse literary styles and genres beyond SF, thereby again necessitating continued human adaptation. This dynamic creates a virtuous circle in the post-digital era ahead, where translators and developers improve LLMs, and improved LLMs, in turn, support them back. Academically, for CBTS, the combined use of MD analysis and functional stylistic analysis has proven effective for comparing human and LLM translations. This methodology offers a robust framework for future studies to examine translator style across different works and genres including but not limited to SF. Ultimately, studies exploring the intersection of linguistics, literature, and artificial intelligence will offer insights into how technology push the boundaries of translation studies and practices, and reshapes the landscape of literary society.

## Data Availability

The datasets presented in this study can be found in online repositories. The names of the repository/repositories and accession number(s) can be found in the article/supplementary material.

## Footnotes

^1^ https://www.douban.com

## References

[ref2] Attebery B. (1992). Strategies of fantasy: Indiana University Press.

[ref3] Attebery B. (2003). *The magazine era: 1926–1960*. *The Cambridge companion to science fiction*. Cambridge: Cambridge University Press, 32–47.

[ref4] Baker M. (2000). Towards a methodology for investigating the style of a literary translator target. Int. J. Transl. Stud. 12, 241–266. doi: 10.1075/target.12.2.04bak

[ref5] Bassnett S. (2013). Translation studies. LondonRoutledge.

[ref6] Biber D. (1991). Variation across speech and writing. Cambridge: Cambridge University Press.

[ref7] Biber D. Conrad S. (2014). Variation in English: Multi-dimensional studies. London: Routledge.

[ref8] Biber D. Conrad S. (2019). Register, genre, and style. Cambridge: Cambridge University Press.

[ref9] Biber D. Finegan E. (1989). Styles of stance in English: lexical and grammatical marking of evidentiality and affect. Text Interdiscipl.J. 9, 93–124. doi: 10.1515/text.1.1989.9.1.93, PMID: 40226361

[ref10] Campbell I. (eds). (2021). Introduction: science fiction and translation. In: Science Fiction in Translation. Studies in Global Science Fiction, (Cham.: Palgrave Macmillan) doi: 10.1007/978-3-030-84208-6_1

[ref12] Cao Y. Zhou L. Lee S. Cabello L. Chen M. Hershcovich D. (2023). Assessing cross-cultural alignment between ChatGPT and human societies: An empirical study. Arxiv. doi: 10.48550/arXiv.2303.17466

[ref13] Čermáková A. (2015). Repetition in John Irving’s novel a widow for one year: a corpus stylistics approach to literary translation. Int. J. Corpus Linguist. 20, 355–377. doi: 10.1075/ijcl.20.3.04cer

[ref14] Chou I. Xiang Z. Liu K. (2024). Unravelling the stylistic nuances: a comparative multidimensional analysis of amateur and professional translations of legends of the condor heroes. Hum. Soc. Sci. Commun. 11, 1–13. doi: 10.1057/s41599-024-03468-6, PMID: 39310270

[ref15] Chu H. Liu S. (2024). Can AI tell good stories? Narrative transportation and persuasion with ChatGPT. J. Commun. 74, 347–358. doi: 10.1093/joc/jqae029

[ref16] Cohen J. (2013). Statistical power analysis for the behavioral sciences. London: Routledge.

[ref18] Csicsery-Ronay I. (2012). The seven beauties of science fiction. Middletown: Wesleyan University Press.

[ref19] Davies M. G. (2004). Multiple voices in the translation classroom: Activities, tasks and projects. Amsterdam and Philadelphia: John Benjamins Publishing.

[ref20] Egbert J. (2012). Style in nineteenth century fiction: a multi-dimensional analysis. Sci. Study Lit. 2, 167–198. doi: 10.1075/ssol.2.2.01egb

[ref22] Freedman C. (2000). Critical theory and science fiction. Middletown, CT: Wesleyan UP.

[ref23] Freud S. (2017). “The uncanny” in Romantic writings. ed. Bygrave S. (London: Routledge), 318–325.

[ref24] Friginal E. Weigle S. (2014). Exploring multiple profiles of L2 writing using multi-dimensional analysis. J. Second. Lang. Writ. 26, 80–95. doi: 10.1016/j.jslw.2014.09.007

[ref25] Gao R. Lin Y. Zhao N. Cai Z. G. (2024). Machine translation of Chinese classical poetry: a comparison among ChatGPT, Google translate, and DeepL translator. Hum. Soc. Sci. Commun. 11, 1–10. doi: 10.1057/s41599-024-03363-0, PMID: 39310270

[ref26] Ghassemiazghandi M. (2024). An evaluation of ChatGPT's translation accuracy using BLEU score. Theory Pract. Lang. Stud. 14, 985–994. doi: 10.17507/tpls.1404.07

[ref27] Goulart L. Staples S. (2023). “Multidimensional analysis” in Conducting genre-based research in applied linguistics. eds. Kessler M. Polio C. (London: Routledge), 127–148.

[ref28] Gray B. (2015). Linguistic variation in research articles: When discipline tells only part of the story. Amsterdam, Netherlands: John Benjamins. doi: 10.1075/scl.71

[ref29] Gu Q. Chen L. (2024). A Corpus-based comparative multidimensional analysis of the two English translations of Luoyang Jialan Ji. Open J. Appl. Sci. 14, 1150–1163. doi: 10.4236/ojapps.2024.144074

[ref30] Harvey J. (2006). Fiction in the present tense. Textual Pract. 20, 71–98. doi: 10.1080/09502360600559795

[ref31] Hatim B. Munday J. (2019). Translation: An advanced resource book for students. London: Routledge.

[ref32] Hearst M. A. (1997). Text tiling: segmenting text into multi-paragraph subtopic passages. Comput. Linguist. 23, 33–64. doi: 10.3115/981732.981734

[ref33] Hunston S. (2010). “How can a corpus be used to explore patterns?” in The Routledge handbook of corpus linguistics. eds. O’Keeffe A. McCarthy M. (London: Routledge), 152–166.

[ref34] Jang M. E. Lukasiewicz T. (2023). Consistency analysis of chatgpt. Arxiv. doi: 10.48550/arXiv.2303.06273

[ref35] Ji M. (2016). “A multidimensional analysis of the translational Chinese genre system” in Corpus methodologies explained. eds. Ji M. Oakes M. Defeng L. Hareide L. (London: Routledge), 53–102.

[ref36] Jiang Z. Lv Q. Zhang Z. Lei L. (2023). Distinguishing translations by human, nmt, and chatgpt: A linguistic and statistical approach. arXiv preprint arXiv:2312.10750.

[ref37] Karpinska M. Iyyer M. (2023). Large language models effectively leverage document-level context for literary translation, but critical errors persist. Arxiv. doi: 10.48550/arXiv.2304.03245

[ref38] Ketterer D. (1971). New worlds for old: the apocalyptic imagination, science fiction, and American literature. Mosaic 47:300. doi: 10.2307/2925508, PMID: 39964225

[ref39] Khoshafah F. (2023). ChatGPT for Arabic-English translation: Evaluating the accuracy. Research Square. doi: 10.21203/rs.3.rs-2814154/v2

[ref40] Leech G. (2007). Style in fiction: A linguistic introduction to English fictional prose. London: Pearson Education.

[ref41] Levin H. (1951). Observations on the style of Ernest Hemingway. Kenyon Rev. 13, 581–609. doi: 10.4159/harvard.9780674424920.c12

[ref42] Luckhurst R. (2005). Science fiction. Cambridge: Polity Press.

[ref43] Mahlberg M. (2013). Corpus stylistics and Dickens’s fiction. London: Routledge.

[ref44] Mahlberg M. McIntyre D. (2011). A case for corpus stylistics: Ian Fleming’s casino Royale. English Text Construct. 4, 204–227. doi: 10.1075/etc.4.2.03mah, PMID: 33486653

[ref45] Mandala S. (2010). The language in science fiction and fantasy: The question of style. London: Bloomsbury Publishing.

[ref46] Mastropierro L. (2018). Corpus stylistics in heart of darkness and its Italian translations. London: Bloomsbury.

[ref47] Nerbonne J. (2008). Variation in the aggregate: an alternative perspective for variationist linguistics. Northern Voices 104, 1162–1164. doi: 10.1353/mlr.2009.0130, PMID: 34409987

[ref48] Nida E. A. (1964). Toward a science of translating: with special reference to principles and procedures involved in bible translating. Leiden: Brill.

[ref49] Nini A. (2015). Multidimensional analysis tagger (version 1.3)-manual. Available at: http://sites.google.com/site/multidimensionaltagger

[ref50] Nini A. (2019). The multi-dimensional analysis tagger. Multi Dimen. Anal. 67:94. doi: 10.5040/9781350023857.0012

[ref51] Noletto I. A. (2024). Fictional languages in science fiction literature: stylistic explorations. New York: Taylor & Francis.

[ref9001] Peng K. Ding L. Zhong Q. Shen L. Liu X. Zhang M.. (2023). Towards making the most of chatgpt for machine translation. arXiv preprint arXiv:2303.13780.

[ref52] Quaglio P. (2009). Television dialogue: The sitcom friends vs. natural conversation. Amsterdam, NL: John Benjamins Publishing Company.

[ref8001] Quirk R. Sidney G. Geoffrey L. Jan S. (1985). A comprehensive grammar of the English language. London: Longman.

[ref53] Ramírez Giraldo J. G. (2019). The limits and forms of literary translation. In Washbourne K. Wyke B. Van The Routledge handbook of literary translation (pp. 8–26). London: Routledge.

[ref9002] Resende N. Hadley J. (2024). The translator’s canvas: Using LLMs to enhance poetry translation. In Proceedings of the 16th Conference of the Association for Machine Translation in the Americas (Volume 1: Research Track) (pp. 178–189).

[ref54] Ryan M. L. (1980). Fiction, non-Factuals, and the principle of minimal departure. Poetics 9, 403–422. doi: 10.1016/0304-422x(80)90030-3

[ref55] Santorini B. (1990). Part-of-speech tagging guidelines for the penn treebank project (3rd revision). Philadelphia: University of Pennsylvania.

[ref57] Su Y. Liu K. (2022). “Orality in translated and non-translated fictional dialogues” in Advances in corpus applications in literary and translation studies. eds. Moratto R. Li D. (London: Routledge), 119–137.

[ref11] Suvin D. Canavan G. (2016). Metamorphoses of science fiction. On the poetics and history of a literary genre. Oxford, United Kingdom: Peter Lang Verlag. doi: 10.3726/978-3-0353-0735-1

[ref58] Tekwa K. (2024). “Artificial intelligence, corpora, and translation studies” in The Routledge handbook of Corpus translation studies. eds. Li D. Corbett J. (London: Routledge), 103–118.

[ref9003] Tomczak M. Tomczak E. (2014). The need to report effect size estimates revisited. An overview of some recommended measures of effect size.

[ref59] Toutanova K. Klein D. Manning C. D. Singer Y. (2003). Feature-rich part-of-speech tagging with a cyclic dependency network. In Proceedings of the 2003 human language technology conference of the north american chapter of the association for computational linguistics (pp. 252–259).

[ref60] Tuzet G. (2022). ‘Suspension of disbelief’: a Coherentist theory of fiction. Int. J. Semiot. Law Revue 35, 455–478. doi: 10.1007/s11196-020-09741-6

[ref61] Wang H. (2023). Defending the last bastion: a sociological approach to the challenged literary translation. Babel 69, 465–482. doi: 10.1075/babel.00330.wan, PMID: 33486653

[ref62] Weldes J. (2003). “Popular culture, science fiction, and world politics: exploring intertextual relations” in To seek out new worlds: Science fiction and world politics. ed. Weldes J. (New York: Palgrave Macmillan US), 1–27.

[ref63] Wolfe G. K. (1986). Critical terms for science fiction and fantasy: A glossary and guide to scholarship. New York: Greenwood Press.

[ref64] Wu K. Li D. Lei V. L. C. (2024). “Corpus-assisted research on translator style” in The Routledge handbook of corpus translation studies. eds. Li D. Corbett J. (Routledge), 271, London–287.

