# Correction: Stylistic variation across English translations of Chinese science fiction: Ken Liu versus ChatGPT

**DOI:** 10.3389/frai.2025.1650320

**Published:** 2025-07-03

**Authors:** Frontiers Production Office

**Affiliations:** Frontiers Media SA, Lausanne, Switzerland

**Keywords:** science fiction translation, large language model translations, corpus-based translation, stylistic variation, Multi-Dimensional analysis

In the acknowledgements statement, the text was incorrectly edited to reflect an additional author without consent. This section has now been removed after a request from the author.

There was a mistake in Table 1 as published. The last row was shifted two places to left adding “101,567” and “99,324” to columns 2 and 3, although these cells should have been blank. The corrected Table 1 appears below.

**Table 1:** Ken Liu's SF translations.

**Author**	**Chinese title**	**English translation and publication year**	**Token totals**						**Segment totals**
			**KL**	**G_1_**	**G_2_**	**G_3_**	**G_4_**	**G_5_**	
Chen Qifan	《鼠年》	*The Year of the Rat*, 2013	9,312	12,379	12,146	12,029	11,876	11,806	74
	《丽江的鱼儿们》	*The Fish of Lijiang*, 2011	5,197	5,485	5,271	5,346	5,258	5,306	39
	《沙嘴之花》	*The Flower of Shazui*, 2012	5,923	5,315	4,981	5,053	4,990	5,190	46
Xia Jia	《百鬼夜行街》	*A Hundred Ghost Parade Tonight*, 2012	6,105	5,579	5,511	5,584	5,466	5,431	47
	《童童的夏天》	*Tongtong's Summer*, 2014	5,718	5,173	5,060	4,997	4,916	4,970	43
	《龙马夜行》	*Night Journey of the Dragon-Horse*, 2016	5,249	4,634	4,960	4,653	4,747	4,641	39
Ma Boyong	《寂静之城》	*The City of Silence*, 2011	14,802	15,467	15,247	15,411	15,302	15,512	109
Hao Jingfan	《看不见的星球》	*Invisible Planets*, 2013	5,969	5,407	5,290	5,430	5,262	5,376	42
	《北京折叠》	*Folding Beijing*, 2015	15,956	14,720	14,186	13,980	13,823	14,024	101
Tang Fei	《黄色故事》	*Call Girl*, 2013	2,767	2,509	2,602	2,588	2,596	2,569	22
Cheng Jingbo	《萤火虫之墓》	*Grave of the Fireflies*, 2014	5,169	4,678	4,737	4,770	4,782	4,637	37
Liu Cixin	《圆》	*The Circle*, 2014	6,885	5,823	6,382	6,250	6,432	6,231	49
	《赡养上帝》	*Taking Care of God*, 2012	12,515	12,155	11,534	11,723	11,863	11,745	92
Total			101,567	99,324	97,907	97,814	97,313	97,438	740

The above works will henceforth be abbreviated as *Rat, Fish, Flower, Ghost, Summer, Journey, City, Planets, Beijing, Girl, Grave, Circle, and God*, respectively in the subsequent tests, tables, and figures.

The original version of this article has been updated.

